# Sarcomatoid (Spindle Cell) Carcinoma of Tongue: A Report of Two Cases

**DOI:** 10.1155/2015/780856

**Published:** 2015-02-17

**Authors:** Montserrat Reyes, Gina Pennacchiotti, Fabio Valdes, Rodrigo Montes, Marcelo Veloso, Maria Angélica Matamala, Luis Zanolli, Gonzalo Rojas-Alcayaga

**Affiliations:** ^1^Department of Pathology and Oral Medicine, Faculty of Dentistry, University of Chile, Sergio Livingstone Pohlhammer 943, Independencia, 8380492 Santiago, Chile; ^2^National Cancer Institute, Profesor Zañartu 1010, Independencia, 8380492 Santiago, Chile

## Abstract

Sarcomatoid Carcinoma (SC) is an unusual and aggressive variant of squamous cell carcinoma, which frequently recurs and metastasizes; for this reason, the right diagnosis is very important. It is considered to be a biphasic tumor made up of cells from squamous and spindle cells carcinoma with a sarcomatous aspect, but of epithelial origin. The diagnosis often represents a clinical-pathological challenge where the study with immunohistochemical technique (IHC) is key to the histopathological diagnosis. The reported cases related to oral mucosa are limited. In this work we present two SC cases where the use of IHC allowed us to achieve a conclusive diagnosis.

## 1. Introduction 

Sarcomatoid Carcinoma (SC) is an unusual morphological variant of squamous cells carcinoma that occurs principally in the upper digestive tract [1, 2]; it consists of a cell proliferation of a squamous carcinoma associated with a malignant spindle stromal compound, but of epithelial origin [3]. Various names have been used to describe Sarcomatoid Carcinoma, such as “fusiform cell carcinoma,” “pleomorphic carcinoma,” “pseudosarcoma,” and “carcinosarcoma,” which reflect the controversy that exists regarding its origin and behavior [4]. It is found more often in the larynx, the nasal cavity, hypopharynx, esophagus, trachea, breast, and oral mucosa [5, 6]. Most of the cases are present in male patients between the sixth and eighth decade of life, with a clinical record associated with alcohol abuse, smoking, and radiation exposure [2, 3, 6].

Due to the fact that SC is an uncommon carcinoma, the histopathological diagnosis is often complex. The histological characteristics that define it include the identification of a poorly differentiated squamous carcinoma, associated with a sarcomatoid transformation, which is being demonstrated by the presence of malignant fusiform cells proliferation [6]. The histogenesis of fusiform cells is controversial. However, it is accepted that it is a monoclonal epithelial neoplasia, which relies on the close association that they have with the squamous carcinoma cells. The studies with immunohistochemical technique (IHC) support the epithelial nature of the mesenchymal component and both neoplasia components possess immunoreactivity for cytokeratin, vimentin, and epithelial membrane antigen (EMA) in most of the cases [7, 8] and the lack of other antibodies, as S-100 or smooth muscle actin alpha [6].

Surgery is the preferred treatment; radiation therapy as well as chemotherapy can be used as a complement to it, but none of them as a separate treatment is recommended as the single therapeutic modality. Generally, the prognosis of this type of carcinomas is not encouraging [6].

The objective of this report is to present two cases of tongue SC with extension to the floor of the mouth and to discuss the diagnostic procedures of a very uncommon malignant neoplasia in the oral cavity.

## 2. Case Report

### 2.1. Case  1

A sixty-year-old male patient was taken to the National Cancer Institute (NCI) with a diagnostic of poorly differentiated squamous tongue carcinoma, with a four-month evolution period, which had lateralized intraoral pain on the left mandibular edge. The patient has personal smoking records of 100 packets per year and he is a heavy alcohol drinker.

**Figure 1:**
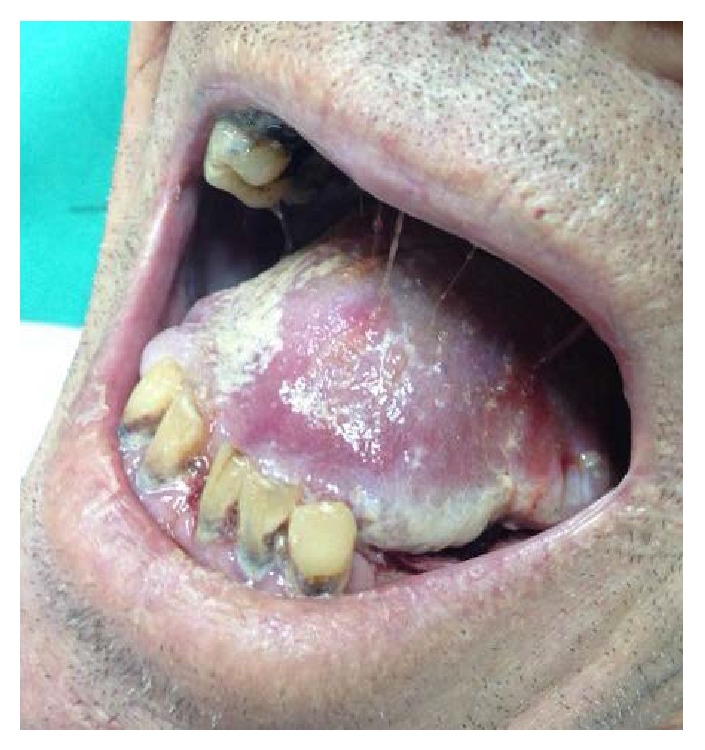
Case one: endophytic tumor lesion with poorly defined margins.

**Figure 2:**
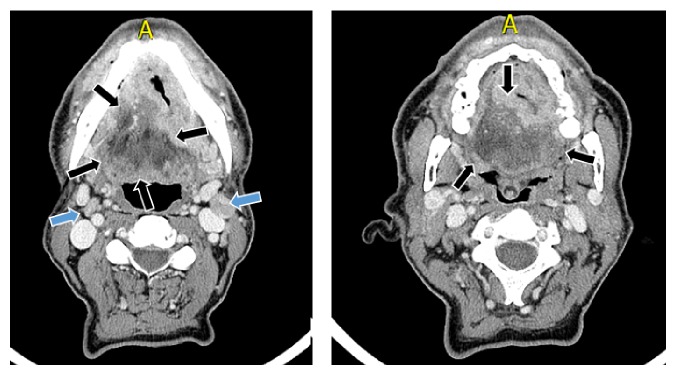
Computed tomography (CT) finding of case one showed that a heterogeneous mass can be seen which spreads to the back of the tongue with extension to the floor of the mouth without reaching the sublingual space (black arrows). The CT confirmed the existence of metastatic lymph nodes (blue arrows).

A clinical examination observed an endophytic growth on the tongue reaching the left jaw and the base of the tongue (Figure 1). Mouth opening was normal. The lesion was classified as T4 N2 M0 and imaging studies were carried out. The neck computerized tomography (CT) confirmed the existence of a lesion of the neoplastic aspect on the floor of the mouth without microscopic evidence of marrow infiltration. The chest CT did not have pathological findings. In maxillofacial CT, as observed in Figure 2, a heterogeneous mass can be seen that spreads to the back of the tongue with extension to the floor of the mouth without reaching the sublingual space.

**Figure 3:**
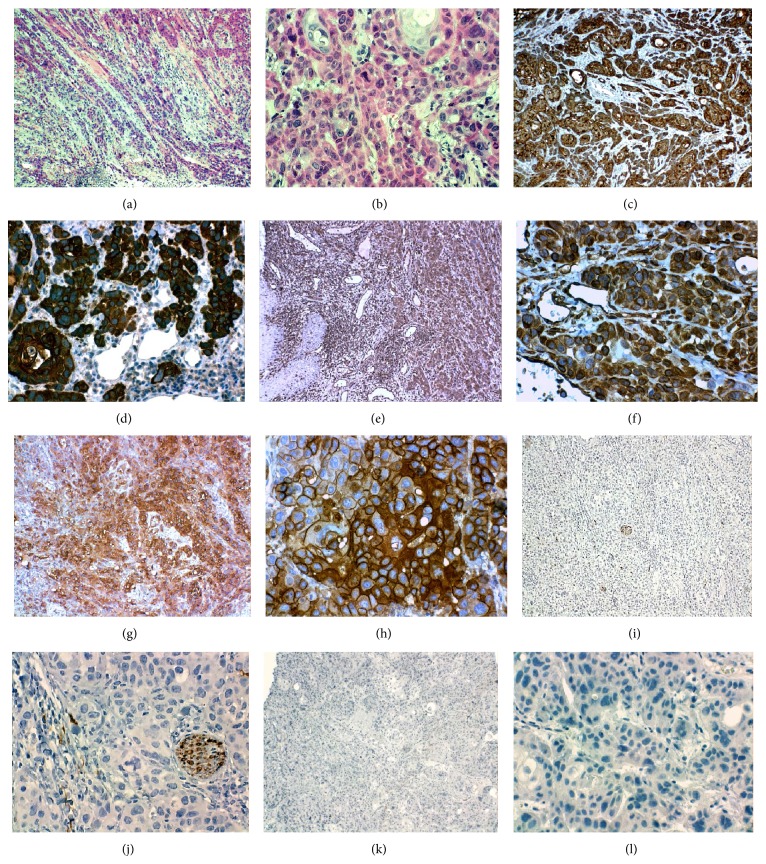
Case one: histopathology of the lesion. (a-b) Hematoxylin-eosin staining. IHC for cytoqueratin AE1/AE3 (c-d); staining of all cells that comprise the neoplasm is shown, as with vimentin (e-f). Expression for EMA (g-h) in epithelial cells. No expression was observed in the neoplastic cells for S-100 (i-j) or smooth muscle actin alpha (k-l).

An oncological resection with jaw resection, bilateral modified radial lymphatic cervical dissection, and mandibular reconstruction with a fibula free flap was carried out. The surgical piece was sent to histopathologic study where the presence of a poorly differentiated squamous carcinoma with marked pleomorphism, cellular atypia, irregular nuclei, and atypical mitosis was reported. Even though the carcinoma cells did not show a fusiform pattern, the carcinoma was much undifferentiated and, with the clinical characteristics of the tumor and the patient's records, this led us to undertake immunohistochemical studies under the suspicion of an unusual variety of oral squamous cell carcinoma (OSCC). The results revealed that the same type of neoplastic cells was positive for cytokeratin AE1/AE3 (Cell Marque laboratory, USA, ready to use), vimentin (Cell Marque laboratory, USA, 1 : 200 dilution), and EMA (Novocastra laboratory, UK, ready to use) and negative for desmin (Cell Marque laboratory, USA, ready to use), S-100 (Cell Marque laboratory, USA, ready to use), and smooth muscle alpha actin (Cell Marque laboratory, USA, 1 : 50 dilution) (Figure 3). Since neoplastic cells do not show spindle pattern, mistakes in the IHC technique, using poorly differentiated OSCC external controls, were discarded, which did not express vimentin in their neoplastic cells, and also the same internal control of the studied sample, because it failed to express vimentin in the remaining epithelium of the mucous; this confirms that there were no mistakes in the technique. Based on the results with IHC, a SC was diagnosed, which was coherent with the records and clinical data of the patient. An adjuvant treatment with radiotherapy and chemotherapy was decided to be performed.

After five months of the surgical procedure, a rapid progression of a pulmonary lesion became evident by clinical examination and radiologically confirmed, which was diagnosed as metastasis. Palliative radiotherapy was indicated.

### 2.2. Case  2

A forty-three-year-old female patient was taken to the NCI with a clinical picture of an ulcerated lesion on the lingual left lateral edge with seven months of evolution that reaches the floor of the mouth. The chest CT did not have pathological findings. The neck CT confirmed the existence of a nodule on the floor of the mouth, whose etiology should be determined by its histopathologic study; also some submental and left jugular lymphs were observed. And incisional biopsy whose histopathological study showed an infiltrating, nonkeratinized moderately differentiated OSCC was carried out. The patient had personal smoking records (7 cigarettes per day) and did not have a history of long-term alcohol abuse.

Once at the NCI, there was a clinical exploration in which an endophytic tumor on the left lateral lingual edge spread to tongue, retromolar base, and trigone. The lesion was classified as T4a N1 M0.

**Figure 4:**
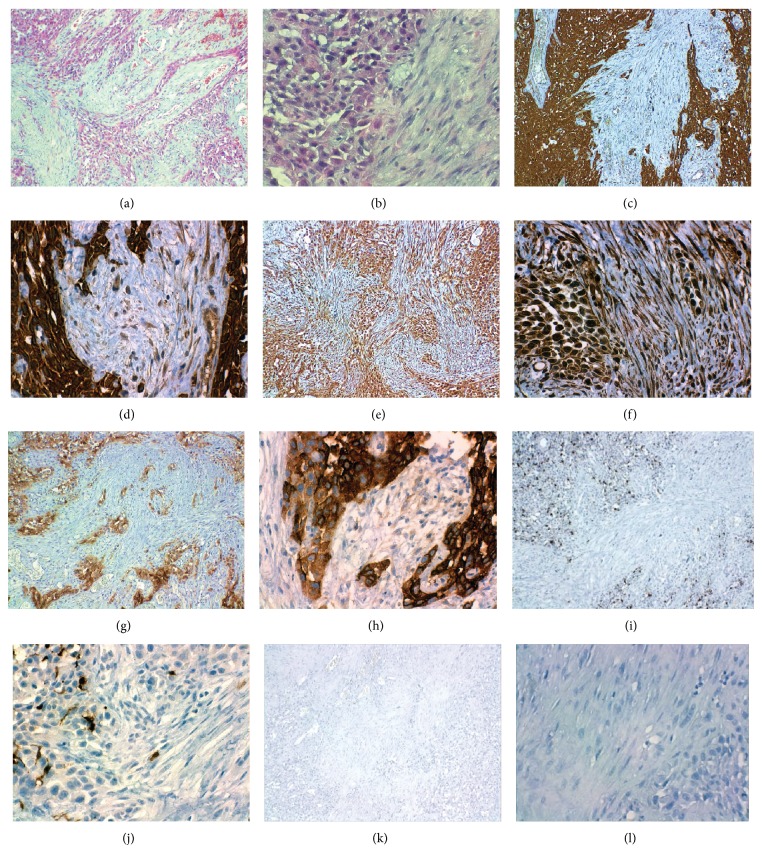
Case two: histopathology of the lesion. (a-b) Hematoxylin-eosin staining of CS. IHC staining for cytokeratin AE1/AE3 (c-d); intense staining was observed in all epithelial cells, and a diffuse staining in spindle cells. Vimentin staining (e-f) shows intense expression of epithelial neoplastic cells and spindle component. EMA intense expression in epithelial cells and weak staining for spindle cells (g-h). No expression was observed in the neoplastic cells for S-100 (i-j) or smooth muscle actin alpha (k-l).

A total glossectomy was carried out through mandibular swing and bilateral modified radial lymphatic cervical dissection. A second biopsy was taken from the tumor and microscopical exam revealed a carcinoma with two neoplastic cellular components, on one hand a proliferation of atypical spindle cells and on the other hand nests of squamous epithelial cells with hyperchromatic nuclei, prominent nucleoli, and atypical mitosis. The studies with IHC revealed that vimentin was strongly positive in the component of spindle cell as in epithelial cells. Cytokeratin AE1/AE3 was strongly positive in epithelial cells and focally positive in spindle cells. EMA antigen was positive for epithelial neoplastic cells and a weak expression in the spindle component was observed. Both neoplasia components were negative for desmin, S-100, and smooth muscle actin alpha (Figure 4). Based on the previous results, a SC was diagnosed. A treatment with postoperative radiotherapy and chemotherapy was indicated. After two months of the surgical procedure, the patient is in good general condition without evidence of metastasis.

## 3. Discussion

The SC is an unusual variant of the squamous cell carcinoma [3]. The SC is made up of cells from a squamous carcinoma which has been poorly differentiated and a stromal component of an epithelial origin; it has been proved that both components have a monoclonal origin and the mesenchymal component would represent an undifferentiation of the squamous component [9].

The SC is predominant in male patients [1] and accounts for less than 2% of all the oral region tumors [3].

In histological terms, it has been reported that the epithelial cells of this tumor undergo progressive phenotypic changes, getting a mesenchymal differentiation in a spindle form, with a mesenchymal matrix component production [2]. The diagnosis of this type of carcinoma is complex and most of the times immunohistochemical studies are needed in order to confirm the expression patterns, where it has been proved that the vimentin and cytokeratin coexpression in the cells that make up the tumor is frequently present and plays a key role in the definite diagnosis [10, 11].

This work reports 2 cases with a tongue SC diagnosis. Although the histology of the first case reported did not show the presence of a spindle pattern, the immunohistochemical technique showed that the cells that make up the tumor were strongly positive for cytokeratin as well as for vimentin and the epithelial cells were positive for the EMA antigen. The suspicion of SC was determined by the low differentiation grade of the neoplasia and the patient's clinical records; therefore in other cases with similar characteristics this type of oral carcinoma should be suspected. References [2, 3, 6] and the immunohistochemical study were considered as a confirmation of the diagnosis. It is important to identify this type of carcinoma because, apparently, it would be a more aggressive variety [6, 9].

The second case reported, unlike the first case, histologically showed both patterns: epithelial and mesenchymal; although one particular feature of this type of tumor is the relative lack of the squamous component [12], in this case, on the contrary, the squamous component prevails over the mesenchymal one. However, the SC diagnosis is not determined by the proportion of some components and even the lack of spindle form component should not exclude the SC diagnosis. The immunohistochemical study is essential, because the histology of the primary tumor is an important parameter which influences selection of initial treatment [13].

Clinically, this type of tumor is characterized by its aggressiveness. Although it is accepted that one of the etiologic agents is the ionizing radiation exposure [9], none of the patients had a radiation exposure record.

SC is potentially aggressive and seems to recur easily and to metastasize. It should be treated accordingly, and better treatment of SC should aim at controlling local and distant recurrence. Surgery is the most well established mode of initial definitive treatment for a majority of oral cancers; their goal is to eradicate the cancer, preserve or restore form and function, minimize the sequelae of treatment, and finally prevent any subsequent new primary cancers. Surgery is preferable in most cases due to the simplicity of the treatment and excellent results with respect to cure and postoperative function [13]. Along with surgical excision, radiotherapy plays a key role in the management of early stage and locally advanced cancer either alone or, more frequently, combined with surgery and/or chemotherapy, which provide an efficient adjuvant treatment, since the surgery as a single treatment is not sufficient and the radiotherapy has to be a mandatory complement to surgery [14, 15]. It has been reported that the progression of this type of tumor is characterized by relapse and metastasis [5, 14]; lungs were the place where metastasis was reported more frequently, according to Thompson et al. [14].

In our work, case 1 meets well the clinical characteristics as well as the described progressions for this neoplasia and case 2 has the histology described for this type of carcinomas without being able to establish the neoplasia aggressiveness yet, taking into account the short amount of time that passed. It is necessary to report in the literature every case of buccal mucosa SC given its limited frequency which makes it difficult to systematically characterize this type of squamous carcinoma.

## 4. Conclusion

The SC is a rare and aggressive variant of the squamous cell carcinoma, whose histogenesis is controversial, and it has, in most cases, a complex diagnosis, due to the histological characteristics of the tumor. That is why the IHC support is essential as well as the understanding of its clinicopathologic characteristics, which is fundamental for the diagnosis and for an appropriate clinical management.
